# Positional RNA-Seq identifies candidate genes for phenotypic engineering of sexual traits

**DOI:** 10.1186/s12983-015-0106-0

**Published:** 2015-07-03

**Authors:** Roberto Arbore, Kiyono Sekii, Christian Beisel, Peter Ladurner, Eugene Berezikov, Lukas Schärer

**Affiliations:** Evolutionary Biology, Zoological Institute, University of Basel, Vesalgasse 1, CH-4051 Basel, Switzerland; D-BSSE, ETH Zürich, Basel, Switzerland; Institute of Zoology and CMBI, University of Innsbruck, Innsbruck, Austria; ERIBA, University of Groningen, University Medical Center Groningen, Groningen, The Netherlands

**Keywords:** Phenotypic engineering, RNA-Seq, RNA interference, simultaneous hermaphrodite, *Macrostomum lignano*

## Abstract

**Introduction:**

RNA interference (RNAi) of trait-specific genes permits the manipulation of specific phenotypic traits (“phenotypic engineering”) and thus represents a powerful tool to test trait function in evolutionary studies. The identification of suitable candidate genes, however, often relies on existing functional gene annotation, which is usually limited in emerging model organisms, especially when they are only distantly related to traditional genetic model organisms. A case in point is the free-living flatworm *Macrostomum lignano* (Lophotrochozoa: Platyhelminthes: Rhabditophora), an increasingly powerful model organism for evolutionary studies of sex in simultaneous hermaphrodites. To overcome the limitation of sparse functional annotation, we have performed a positional RNA-Seq analysis on different body fragments in order to identify organ-specific candidate transcripts. We then performed gene expression (*in situ* hybridization) and gene function (RNAi) analyses on 23 candidate transcripts, both to evaluate the predictive potential of this approach and to obtain preliminary functional characterizations of these candidate genes.

**Results:**

We identified over 4000 transcripts that could be expected to show specific expression in different reproductive organs (including testis, ovary and the male and female genital systems). The predictive potential of the method could then be verified by confirming organ-specific expression for several candidate transcripts, some of which yielded interesting trait-specific knock-down phenotypes that can now be followed up in future phenotypic engineering studies.

**Conclusions:**

Our positional RNA-Seq analysis represents a highly useful resource for the identification of candidate transcripts for functional and phenotypic engineering studies in *M. lignano*, and it has already been used successfully in several studies. Moreover, this approach can overcome some inherent limitations of homology-based candidate selection and thus should be applicable to a broad range of emerging model organisms.

**Electronic supplementary material:**

The online version of this article (doi:10.1186/s12983-015-0106-0) contains supplementary material, which is available to authorized users.

## Introduction

Methods to experimentally generate phenotypic variation in evolutionary studies traditionally include experimental evolution, environmental manipulation, and direct trait manipulation [1, 2]. Among these methods, the last one-often referred to as phenotypic engineering-offers the greatest potential to manipulate the phenotypic value of a trait towards extreme values, often above and below the natural range, thus enhancing the statistical power to detect how selection acts on specific traits. Moreover, phenotypic engineering has the advantage of specifically targeting the trait of interest, thereby minimizing confounding effects of experimentally induced variation in other traits. Examples include the mechanical modification of morphological and functional traits (e.g. [3–6]), as well as physiological and behavioural modification using hormones (e.g. [7]) or different dietary treatments (e.g. [8]). More recently, genetic engineering has opened up the possibility of manipulating the molecular basis of traits [9]. While the scope for transgenesis was previously restricted to model organisms with established genetic and genomic tools, recent genome editing technologies, such as TALEN and CRISPR/Cas-based methods, are currently being established in many emerging model organisms (e.g. [10–12]).

Alternative tools to alter molecular functions are gene expression knock-down methods [13], the gold standard of which is RNA interference (RNAi; e.g. [14–16]). The combination of its cost effectiveness, scalability and broad applicability makes this method accessible for evolutionary studies in a growing range of species [17]. RNAi enables the modification of normal gene expression to produce phenotypes that mirror the effect of reduced- or even loss-of-function mutations, allowing functional and genetic studies of specific genes. In the context of phenotypic engineering, the great potential of RNAi is based on its ability to target (i) internal structures (organs or cells) that are inaccessible to mechanical modification and (ii) phenotypes that are influenced by the function of single genes, thus reducing the risk of manipulating several traits at once. The application of this method for evolutionary and ecological studies is still largely restricted to traditional model organisms (e.g. [18, 19]) with some exceptions in non-model organisms (e.g. [20, 21]). An interesting recent example in a non-model organism is the study of Khila et al. [22], who reported a reduction in the reproductive success of water strider males with reduced antennal elaborations, which result from RNAi knock-down of the highly conserved gene *distal-less* (*Dll*). The study demonstrated the adaptive role of male antennal elaborations in grasping the female during pre-mating struggles and suggested that sexual conflict drove the evolution of a novel male-specific function of *Dll*, which is involved in the development of this sexually antagonistic trait. But despite this highly encouraging recent example, RNAi based phenotypic engineering to experimentally test evolutionary predictions in emerging model organisms has yet to reach its full potential.

One reason for this slow progress is that-while protocols for RNAi are becoming available for an increasing number of species (e.g. *Hydra* [23, 24]; *Tribolium*, [25]; *Daphnia* [26]; sponges [27]; and *Platynereis* [28])-the selection of candidate genes for knock-down still poses a significant challenge in most emerging model organisms. Candidates might be selected based on *a priori* knowledge of their function, which is often missing since direct experimental gene annotation is necessarily limited in emerging model organisms [29]. A commonly adopted alternative approach for candidate gene selection is therefore comparative functional genomics, where putative functionally conserved genes associated with a given phenotype are identified in related model species (as in the water strider study above). However, the usefulness of this approach greatly depends on the phylogenetic distance between the respective study species [30]. Moreover, this approach is particularly problematic for reproduction-specific genes (and especially genes with male-biased expression), because these tend to evolve rapidly and often diverge to a point where their homology to other genes cannot anymore be recognized. For example, genes with male-biased expression show a substantially lower fraction of identifiable orthologs between *Drosophila* species than genes with female-biased or unbiased expression [31, 32]. Finally, comparative candidate gene selection also suffers from severe biases in species coverage of well-annotated genomes [30]: traditional genetic model organisms belong predominantly to the superphyla Ecdysozoa (e.g. *Drosophila* and *Caenorhabditis*) and Deuterostomia (e.g. *Danio rerio* and *Mus musculus*). Despite the recent emergence of a few molecular model organisms among the Lophothrochozoa [28, 33–36], extensive functionally-annotated sequence data is still missing in this clade, reducing the power of candidate gene approaches based on sequence homology to identify RNAi targets in species belonging to this superphylum.

These problems apply in our evolutionary research on the reproductive biology of the free-living flatworm *Macrostomum lignano* (Lophotrochozoa: Platyhelminthes: Rhabditophora) [37]. While the emphasis of our own work has been on empirical tests of predictions from sexual selection (e.g. [38–40]), sexual conflict (e.g. [41, 42]) and sex allocation theory (e.g. [43, 44]) the research in the *Macrostomum* community as a whole also encompasses stem cell biology (e.g. [45–48]), regeneration (e.g. [49, 50]), aging [51], and germ cell biology and gametogenesis (e.g. [52, 53]). This has led to the establishment over the last several years of gene expression and function analysis tools such as *in situ* hybridization (ISH) [47] and efficient RNAi by soaking [54]. The availability of these powerful experimental techniques and the growing understanding of its reproductive biology thus make *M. lignano* a highly amenable system in which to use RNAi-based phenotypic engineering to address evolutionary questions.

Recently, Sekii et al. [55] adopted a dose-dependent RNAi method to quantitatively manipulate sperm production rate and-probably as a consequence-copulation frequency, observing significant positive correlations between these traits and paternity success, as predicted by sperm competition theory. The candidate gene for that study resulted from a classical homology-based candidate gene approach, which identified *macbol1* as a highly conserved member of the *boule* gene family [52]. However, the same screen also revealed that many other transcripts that showed reproduction specific annotations in classical models where not reproduction-specific in *M. lignano* (K. Sekii, personal observation), thus severely limiting the number of suitable candidate genes and highlighting the limitations of this approach.

In this study, we therefore decided to apply a ’positional’ RNA-Seq strategy directly in *M. lignano*, with the aim of identifying candidate transcripts for phenotypic engineering independently of any prior sequence annotation. To this end, we sequenced the transcriptomes of four samples obtained by cutting worms at different levels along the anterior-posterior body axis, broadly corresponding to the boundaries between the head, testis, ovary and tail regions of the worm. By comparing levels of expression of transcripts in these samples, we obtained a prediction of their site of expression, and thus a list of candidate transcripts likely to be enriched for genes that function specifically in different reproductive organs. An ISH screen of 23 selected candidate transcripts successfully identified many organ-specific transcripts, thus confirming the predictive potential of the positional RNA-Seq data. Finally, an RNAi screen identified five evident knock-down phenotypes, some of which represent valuable candidates for future phenotypic engineering studies in *M. lignano*.

## Results

### Positional transcriptome

**Fig. 1:**
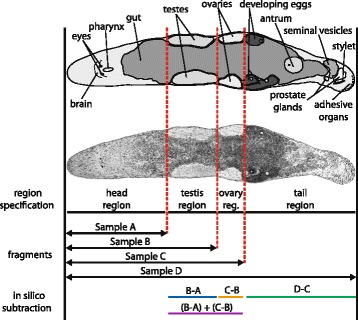
Anatomy of *Macrostomum lignano* and sampling design for RNA-Seq. The main organ systems reside in four regions along the anterior-posterior body axis (i.e. the head, testis, ovary and tail regions). By cutting the worms in between two body regions (red dotted lines), four samples for RNA-Seq were collected (two-headed arrows): Sample A (head region; n = 400 worms); Sample B (head and testis regions; n = 300 worms); Sample C (head, testis and ovary regions; n = 200 worms); Sample D (entire worm, i.e. head, testis, ovary and tail regions; n = 100 worms). Differences in expression between adjacent samples were scored in order to classify the transcripts (in silico subtraction; see Table 1). Adult worms are about 1.5 mm in length. Interference contrast micrograph modified from Schärer et al. (2007) [97]

We defined four regions along the anterior-posterior body axis of *M. lignano* (Fig. 1). The head region contains the rostrum, eyes, brain and pharynx with associated glands. The pharynx opens into the gut that stretches along almost the entire animal. In the testis and ovary regions the space on both sides of the gut is primarily occupied by paired testes and ovaries, respectively. Finally, the tail region usually contains developing eggs, the female and male genitalia, and the tail plate with its adhesive organs. The female genitalia consist of the female antrum, which stores the received sperm after copulation and serves as an egg laying organ, surrounded by shell and cement glands. The male genitalia are located posterior to the antrum and consist of the false seminal vesicle and the muscular true seminal vesicle (both containing sperm ready for donation), the prostate gland cells (producing seminal fluid), and the stylet (male copulatory organ).

**Fig. 2:**
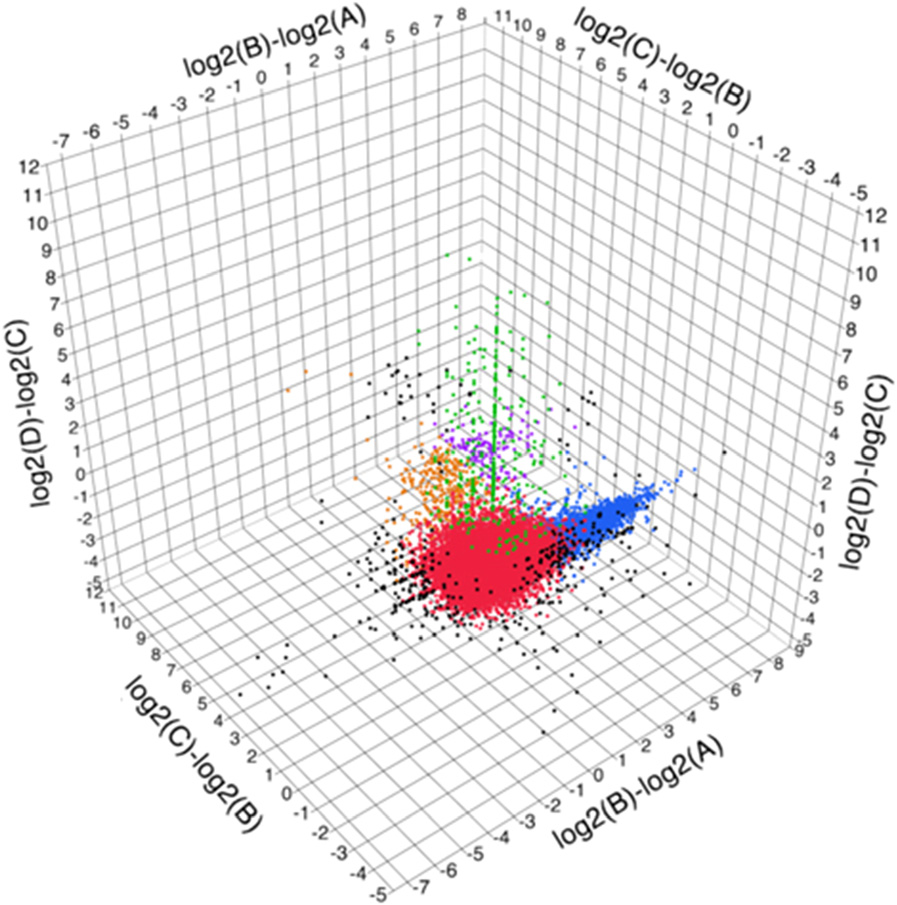
Positional transcriptome of *Macrostomum lignano*. Differences in log2-expression level of the transcripts between adjacent RNA-Seq samples (i.e. B vs. A, C vs. B and D vs. C; see Fig. 1 for information about the different samples and Methods for details on the calculation of the expression level). The colours highlight different classes of transcripts (see Table 1 and Methods): non-differentially expressed transcripts (red); testis region-specific transcripts (blue); ovary region-specific transcripts (purple and orange); tail region-specific transcripts (green); others (black). See Additional file 2 for an animated version of this figure

By cutting many animals at one of the three levels delineating the borders between the four body regions (Fig. 1, for details see Methods), we collected four samples for RNA-Seq differing in their tissue composition. Specifically, the different samples contained either (A) the head region, (B) the head and testis regions, (C) the head, testis and ovary regions or (D) the head, testis, ovary and tail regions (i.e. entire worms). The rationale behind this sampling strategy was that cut fragments that missed the chosen cutting level were discarded and cutting twice would have greatly increased such losses. Between 56 % and 69 % of the generated Illumina reads from the four different libraries could be mapped to 74708 transcripts, corresponding to 97.7 % of the whole reference transcriptome (version MLRNA110815; [56, 57]). We then compared the expression level of each transcript between adjacent samples in order to identify transcripts with expression profiles that suggest specific expression in the different body regions (Table 1; Fig. 2; Additional file 1; Additional file 2; log2-expression level differences higher than 2 were considered indicative of differential expression). 93.7 % of the transcripts showed no differential expression between samples. The remaining 6.3 % showed differential expression between two or more adjacent samples. To classify the transcripts we used a three-digit code referring to the comparison between pairs of adjacent samples (i.e. [B vs. A, C vs. B, D vs. C]; the symbols “+” and “0” define differential expression and no differential expression respectively; see Table 1 and Methods). The majority of the differentially expressed transcripts grouped into four classes representing transcripts putatively specific for the testis (class [+,0,0]), ovary (classes [+,+,0] and [0,+,0]), and tail (class [0,0,+]) regions (Table 1). Note that we include the [+,+,0] class in the putatively ovary-specific transcripts because the close spatial proximity of the testes and ovaries may have led to some contamination of ovarian tissue in the testis-fragment (see also Discussion).

**Table 1:** Positional classification of the transcriptome of *Macrostomum lignano* and details on the candidate transcripts selected for validation

Class	n	%	Mean B-A	Mean C-B	Mean D-C	Candidates	B-A	C-B	D-C	ISH	RNAi
[0,0,0]	70064	93.7	−0.14	−0.09	0.09	RNA815_92.1	1.15	0.55	0.24	X	-
Non-diff.						RNA815_40.1	0.82	0.27	0.51	X	-
expressed						RNA815_2403.2	−1.08	−0.23	−0.32	X	X
						RNA815_2224.1	0.52	−0.02	0.24	-	-
[+,0,0]	3360	4.4	4.00	−0.18	−0.41	RNA815_7008	5.17	−0.03	−0.39	X	X
Testis region						RNA815_9973.1	5.02	−0.03	−0.41	X	X
						RNA815_6628.2	4.13	−0.27	−0.46	X	-
						RNA815_3228	4.50	−0.25	−0.67	X	-
						RNA815_10311.2	2.89	0.61	−0.14	X	-
						RNA815_9262	2.55	0.12	−0.24	X	-
[+,+,0]	127	<0.1	3.18	4.14	0.52	RNA815_16738	2.77	4.75	0.77	X	-
Ovary region						RNA815_1618.1	5.46	4.35	0.47	X	-
						RNA815_2640	3.62	4.40	0.67	X	X
						RNA815_7725.2	2.91	4.15	−0.01	X	-
						RNA815_7498	2.28	4.92	0.61	X	-
						RNA815_12337.1	2.32	6.04	−0.14	-	-
[0,+,0]	323	0.4	0.57	3.41	0.36	RNA815_4558	0.39	4.09	0.64	X	-
Ovary region						RNA815_6266	2.00	5.35	0.88	-	-
[0,0,+]	366	0.4	−0.02	−0.03	4.23	RNA815_22046	0	1.00	7.69	X	-
Tail region						RNA815_80.4	0	0	8.30	X	X
						RNA815_9549.4	0	0	8.93	X	-
Others	468	0.6				RNA815_5404.2	3.58	−0.18	3.97	X	-
						RNA815_13	0	4.85	4.87	X	-

The transcripts are classified on the basis of their differential expression profile between samples using a three digit code (the 'Class'). Higher than two-fold differences in log2-expression level were considered indicative of differential expression (see Methods). Each digit in the code refers to the comparison between two adjacent samples (i.e. [B vs. A, C vs. B, D vs. C]) with the following coding: positive differential expression (+), no differential expression (0) or negative differential expression (−). The classes correspond to the predicted expression in different organs and body regions. We also give the number (n) and percentage (%) of transcripts in each class and the class mean difference in log2-expression between samples (Mean B-A, Mean C-B, Mean D-C). Furthermore, we give the accession codes of the selected candidate transcripts belonging to different classes (Candidates), the transcript difference in log2-expression between samples (B-A, C-B, D-C), and the summary of the *in situ* hybridization (ISH) and RNA interference (RNAi) screens, namely expression or phenotype detected (X) or non-detected (−)

### *In situ* hybridization screening

We performed an ISH screen to test whether our positional transcriptome could successfully predict the site of expression of the transcripts. 23 candidates were chosen, independently of any previous sequence annotation, spread over different classes (Table 1, Additional file 3, see Additional file 4 for gene annotation), namely non-differentially expressed transcripts (class [0,0,0], n = 4), testis-region specific transcripts (class [+,0,0], n = 6)*,* ovary-region specific transcripts (class [+,+,0], n = 6; class [0,+,0], n = 2)*,* tail-region specific transcripts (class [0,0,+], n = 3), and two other classes (class [+,0,+], n = 1 and class [0,+,+]), n = 1).

**Fig. 3:**
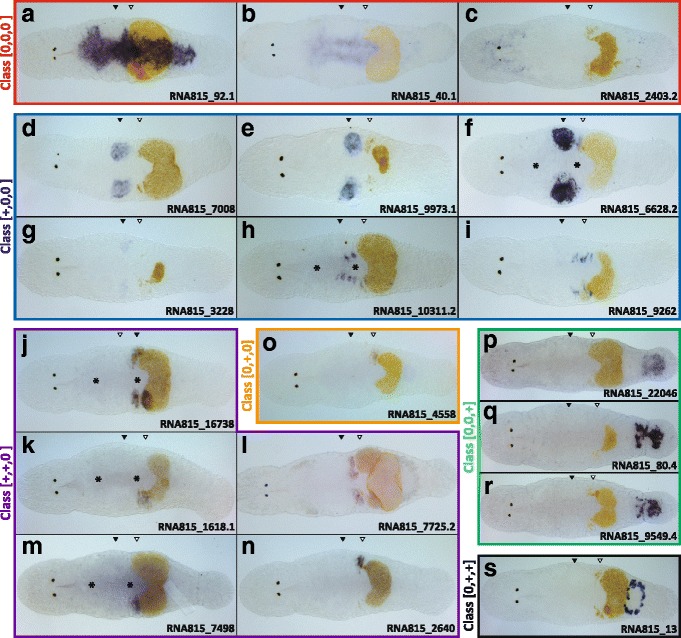
*In situ* hybridization (ISH) screen of positional candidate transcripts in *Macrostomum lignano*. ISHs of transcripts of different classes (see main text and Table 1). (**a**-**c**) Class [0,0,0]: non-differentially expressed transcripts (red box). (**d**-**i**) Class [+,0,0]: testis region-specific transcripts (blue box). (**j**-**n**) Class [+,+,0]: ovary region-specific transcripts (purple box). (**o**) Class [0,+,0]: ovary-region specific transcripts (orange box). (**p**-**r**) Class [0,0,+]: tail region-specific transcripts (green box). (**s**) Class [0,+,+]: belonging to “other classes” (black box). Note the level of the testes (black triangles) and ovaries (white triangles), and the nonspecific background staining (*). Eggs at advanced developmental stages appear yellow or orange in the specimens

**Fig. 4:**
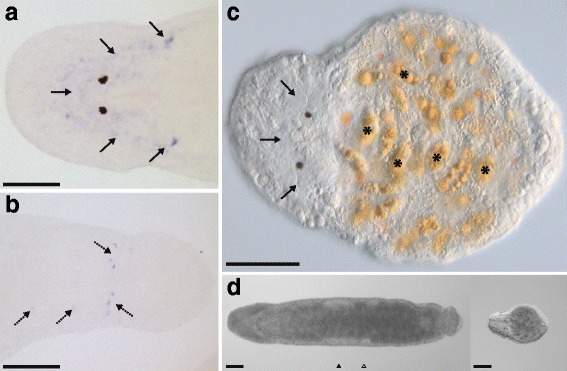
RNAi phenotype and ISH pattern of the [0,0,0] class candidate RNA815_2403.2. **a** Control ISH pattern in the head region with stained cells (solid arrows). (**b**) Control ISH pattern in the tail region with putative neuronal clusters (dashed arrows). (**c**) Interference contrast micrograph of a knock-down worm. (**d**) Overall appearance of a control (left) and a knock-down (right) worm. Note the level of the testes (black triangle) and the ovaries (white triangle); and the algae in the gut (*); Bars = 100 μm

Among the four non-differentially expressed candidates (class [0,0,0]), two showed specific expression in the gut (Fig.3a-b), while another was expressed in both the head and tail regions (Figs.3c and 4a-b). In the head region, the distribution of the stained cells broadly overlaps with the brain (anterior to the eyes, see Fig. 1). In the tail region, the distribution of the stained cells resembles that of neuronal clusters described by Ladurner et al. [58]. These results potentially suggest specific expression of this candidate (RNA815_2403.2) in the nervous system. No expression was observed for RNA815_2224.1 (not shown).

**Fig. 5:**
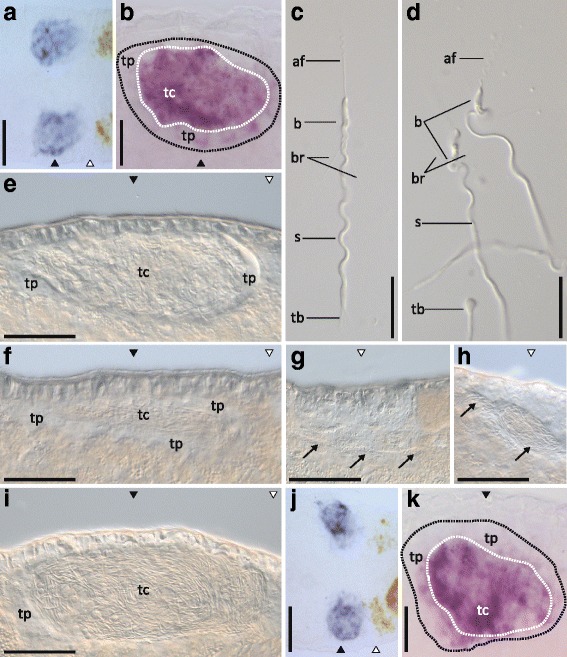
RNAi phenotype and ISH pattern of the [+,0,0] class candidates RNA815_7008 and RNA815_9973.1. **a** Control ISH pattern of RNA815_7008 in the testis region. (**b**) Detail of control ISH pattern of RNA815_7008 in the testis. (**c**) Control sperm. (**d**) RNA815_7008 knock-down sperm. (**e**) RNA815_7008 knock-down testis. (**f**) Control testis. (**g**) Control vas deferens in the ovary region. (**h**) RNA815_9973.1 knock-down vas deferens in the ovary region. (**i**) RNA815_9973.1 knock-down testis. (**j**) Control ISH pattern of RNA815_9973.1 in the testis region. (**k**) Detail of control ISH pattern of RNA815_9973.1 in the testis. Black and white dotted lines delimit the testis and the testis centre respectively. Note the level of the testes (black triangles) and the ovaries (white triangles). Testis periphery (tp); testis centre (tc); sperm anterior feeler (af), body (b), bristles (br), shaft (s) and terminal brush (tb); vas deferens (arrows). Bars = 50 μm (**A**, **C**, **D**, **E**, **H**, **I**, **J**), 30 μm (B, K), 20 μm (**F**, **G**)

Four of six testis-region specific candidates (class [+,0,0]) were indeed expressed exclusively in the testis (Fig.3d-g). The expression appeared to be limited to the testis centre, where spermatids and mature sperm are located, while little or no expression could be detected in the peripheral regions, where spermatogonia and spermatocytes I and II are located [54] (see Fig. 5a-b and j-k for more detailed ISHs of RNA815_7008 and RNA815_9973.1, two candidates for which we found RNAi phenotypes). The other two candidates showed specific expression in a sub-population of cells in the gut at the level of the testis region, but also to a lesser degree at the level of the ovary region (Fig.3h-i).

**Fig. 6:**
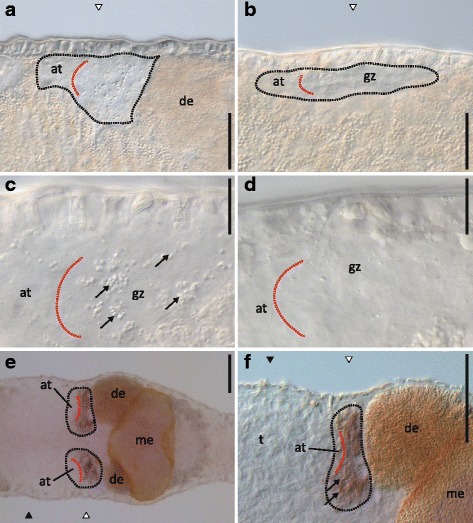
RNAi phenotype and ISH pattern of the [+,+,0] class candidate RNA815_2640. **a** Control ovary. (**b**) knock-down ovary. (**c**) Detail of the control ovary. (**d**) Detail of the knock-down ovary. (**e**) ISH pattern in the ovaries. (**f**) Detail of the ISH pattern in the ovary. Black dotted lines delimit the ovary. Red dotted line approximately divide the anterior tip and the growth zone of the ovary. Level of the testes (black triangles) and the ovaries (white triangles); anterior tip (at) and growth zone (gz) of the ovary; testis (t), developing eggs (de); mature egg (me); yolk and eggshell granules in the ovary (arrows). Bars = 50 μm (A, B, E, F), 20 μm (C, D)

Five of the six ovary-region specific candidates belonging to class [+,+,0] were expressed in ovaries (Fig. 3 j-n) (while no expression was observed for the sixth candidate, RNA815_12337.1). Moreover, one of the two ovary-region specific candidates belonging to class [0,+,0] showed specific expression in the ovaries (Fig. 3o) (while no expression was observed for the other candidate, RNA815_6266). The ISH patterns were largely consistent for all of these candidates, with the expression being localized in the growth zone of the ovaries and often also in the developing eggs (e.g. Fig. 3 j). Note that Fig. 6e-f shows more detailed ISHs of one candidate for which we found an RNAi phenotype (RNA815_2640).

**Fig. 7:**
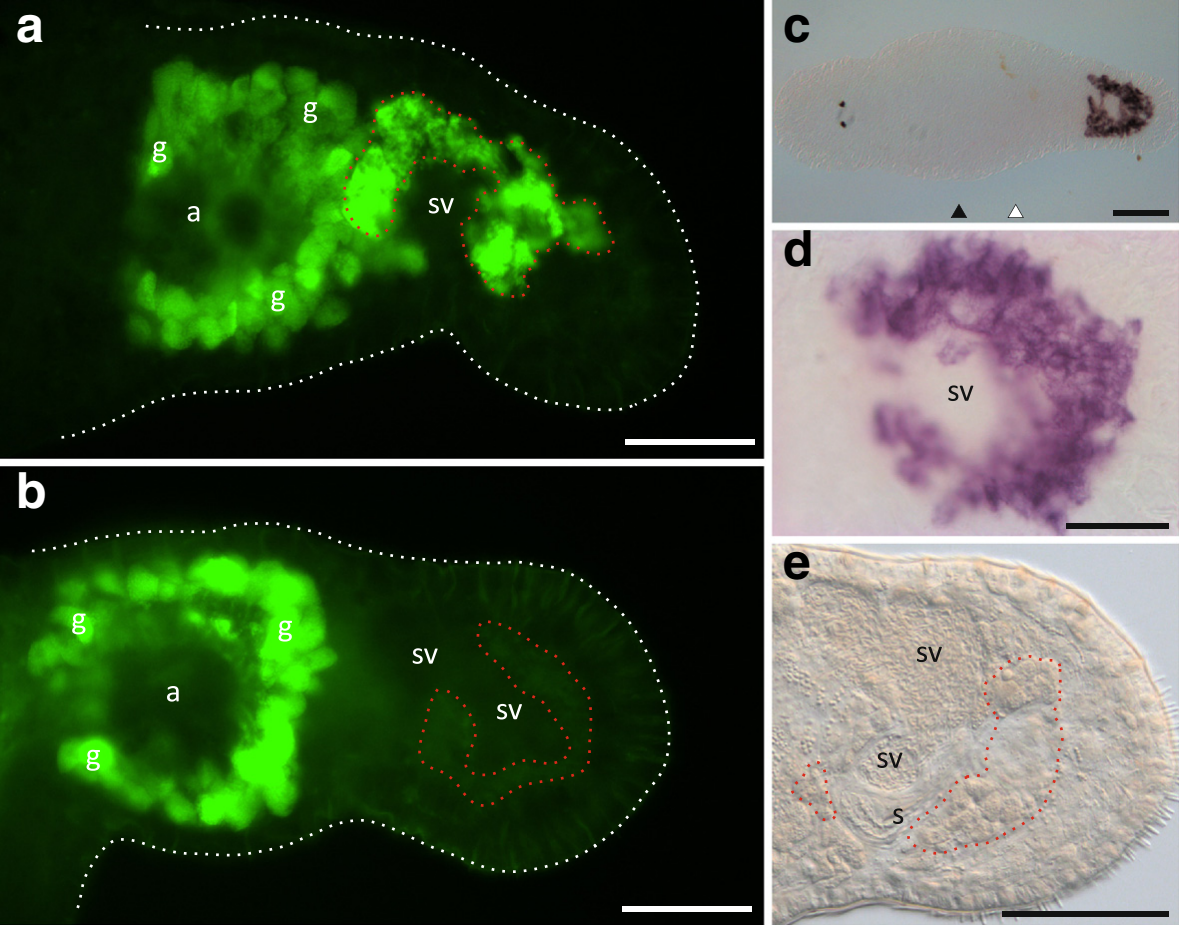
RNAi phenotype and ISH pattern of the [0,0,+] class candidate RNA815_80.4. Prostate gland-specific antibody (MPr-1) staining in the tail region of (**a**) a control worm and (**b**) a knock-down worm. Note the non-specific antibody staining of the shell/cement glands (**g**) surrounding the female antrum (**a**). (**c**) Whole mount ISH pattern. (**d**) Detail of the ISH pattern in the prostate glands. (**e**) Detail of the posterior portion of the tail region of a knock-down worm. White dotted lines show the contour of the animals. Red dotted lines highlight the approximate region of the prostate gland cell bodies. Level of the testes (black triangles) and the ovaries (white triangles); shell/cement glands (g); seminal vesicles (sv); stylet (s). Bars = 100 μm (C), 50 μm (A, B, E), 20 μm (D)

All three tail-region specific candidates (class [0,0,+]) showed specific expression in the prostate glands (Fig. 3p-r), and the expression pattern was similar for the three transcripts. Figure. 7c-d shows the ISH staining of RNA815_80.4 clearly confined to the bodies of the prostate glands that surround the seminal vesicles.

Finally, the candidate belonging to class [+,0,+] provided only a weak ISH signal in the prostate glands (data not shown) and the candidate of class [0,+,+] was expressed in the shell and cement glands that surround the female antrum in the tail region (Fig. 3s).

### RNAi screening

In order to obtain preliminary functional characterizations of the different candidate transcripts, we determined their RNAi knock-down phenotypes by soaking the worms with transcript-specific dsRNA from the first day post-hatching until the control animals had reached sexual maturity. During the screen, phenotypic effects were documented *in vivo* by interference contrast microscopy and, for some transcripts, immunocytochemistry (for details see Methods). We detected phenotypic effects of the RNAi treatment for five candidates (RNA815_2403.2, RNA815_7008, RNA815_9973.1, RNA815_2640 and RNA815_80.4; see Table 1). The observed RNAi phenotypes were consistent among replicates (Additional file 5) as previously reported for *M. lignano* (e.g. 53, 54). No ISH signal could be detected after the RNAi treatment for RNA815_7008, RNA815_9973.1, RNA815_2640 and RNA815_80.4 (Additional file 5, Additional file 6). Although functional or ultrastructural effects of the RNAi treatment for other candidates cannot of course be excluded, we here focus on the description of these five evident knock-down phenotypes and briefly mention functional studies of orthologous genes in other species, where available.

#### RNA815_2403.2 is required for prohormone processing during postembryonic development

In control animals, the ISH pattern of RNA815_2403.2 suggests an expression that is specific to the nervous system (Fig. 4a-b), although the distribution of the stained cells does not overlap with any previously described functional classification of the nervous system in *M. lignano*, i.e. neither with the staining of the serotonergic and GYIRFamidergic nervous system [59] nor with the FRMFamidergic nervous system (P. Ladurner, unpublished data). Knock-down of RNA815_2403.2 resulted in dramatic effects on postembryonic development: after one week of dsRNA treatment the worms were smaller than the controls, showed a roundish body shape and failed to produce any reproductive organs (Fig. 4c-d). All worms died after 12–14 days of treatment. Sequence annotation analysis identified RNA815_2403.2 as a member of the peptidase S8 protein-convertase family and as a homolog of *Smed-prohormone convertase 2* (*Smed-pc2*, DAA33932, Additional file 4). *Smed-pc2* is expressed in the central nervous system in the planarian *Schmidtea mediterranea* [60] and is essential for the processing of several prohormones into mature peptide hormones [61]. RNAi knock-down of *Smed-pc2* results in abnormalities in movement coordination, regeneration and the development of the reproductive system, revealing the importance of prohormone processing in various neural and physiological functions in flatworms. We hypothesise a similar prohormone processing function of RNA815_2403.2 in *M. lignano* and conclude that the severe consequences of its knock-down possibly reflect effects of prohormone processing failure during postembryonic development. The efficiency of the RNA815_2403.2 knock-down could not be assessed by ISH as the animals were followed until death.

#### The knock-down of RNA815_7008 affects spermiogenesis

In *M. lignano*, the testes mainly consist of male germ cells at different developmental stages [52, 54, 61]. The peripheral layer contains spermatogonia and spermatocytes I and II and the testis centre is formed by maturing spermatids and mature sperm. ISH revealed that the expression of RNA815_7008 was strong in the basal layer of the testis centre (where maturing spermatids are located), while being weak or absent in the periphery, indicating a role of this gene in late male germ cell development (Fig. 5a-b). The knock-down of the testis-specific transcript RNA815_7008 yielded fully developed adults that nevertheless exhibited an aberrant sperm and testis morphology (Fig. 5d-e). In the dsRNA treatment, a relatively normal overall testis structure and size were established during sexual development, but the testis centre showed a disorganized arrangement of the elongated spermatids (mid-and late-spermatids) and of the mature sperm (Fig. 5e), which, is in contrast to the orderly parallel organization in control testes (Fig. 5 f).

Interesting knockdown effects were also observed on the intriguing sperm morphology of this flatworm. The sperm of *M. lignano* are elongated and consist of a body with an anterior feeler and a posterior shaft, followed by a terminal brush [37, 42] (Fig. 5c). A pair of lateral bristles pointing backwards is found at the junction between the body and the shaft. The complex sperm morphology is the result of a series of developmental events during spermatogenesis, where spermatids undergo several changes at the structural and ultrastructural level [42, 62]. The knock-down sperm released from the seminal vesicles (Fig. 5d) displayed all the components of the control sperm. However, at the junction between the body and the shaft, where the bristles emerge, the sperm presented a coil-like appearance that led to a twisting of the sperm. The lateral bristles of most of the knock-down sperm observed were oriented forwards, as is also observed in late control spermatids [37], and they appeared more flexible than those of the control sperm. Moreover, in the knock-down sperm, the terminal brush often appeared as a roundish structure reminiscent of the residual body of late control spermatids [37].

The specific expression of RNA815_7008 in the testis centre, the overall morphology of the knock-down sperm, and in particular the bristle orientation and terminal brush morphology, suggest an essential function of this gene for spermatid maturation during the very late phases of spermatogenesis. Gene annotation analysis (Additional file 4) revealed RNA815_7008 as a Serine/threonine-protein phosphatase PP1-beta catalytic subunit 1 (PP1), but with a homology to an "unnamed protein product" in mouse (BAB23473.1) and humans (BAH14903.1).

#### The knock-down of RNA815_9973.1 affects the male reproductive system

The knock-down phenotype of another testis-specific gene, RNA815_9973.1, was evident in the testis, in the seminal vesicles and in the vas deferens. The expression pattern of RNA815_9973.1 was similar to that of RNA815_7008, with specific expression in the testis centre (Fig. 5j-k). The testes of treated worms appeared larger than normal, with a thin peripheral layer and an expanded lumen full of elongated spermatids and mature sperm (Fig. 5i), and lacking the usual parallel organization found in control testes (Fig. 5 f). In two cases, at the level of the ovaries, the worms presented one or two bulges in the vas deferens (Fig. 5h). Such structures were never observed in our control worms (Fig.5g) and they appear to be formed by the accumulation of sperm in the vas deferens. The seminal vesicles often appeared relatively empty, but the mature sperm found there appeared to show normal morphology and behaviour (data not shown). Sequence annotation of RNA815_9973.1 did not produce any significant result (Additional file 4).

#### CPEB (RNA815_2640) mediated translational control is essential for oocyte maturation

The knock-down of the ovary-specific transcript RNA815_2640 drastically affected the female reproductive function and resulted in the complete inability of the worms to develop mature eggs (Fig. 6). In control worms the ovary is a relatively weakly organized structure consisting of an anterior tip that is mainly formed by oogonia and of a growth zone where oocytes I at different developmental stages are located (Fig. 6a). During oogenesis, egg shell and yolk granules are deposited in the cytoplasm of oocytes I that have started to develop into eggs (Fig. 6c) [37]. Maturing eggs continue to develop as they leave the ovary, while building up increasing amounts of yolk and egg shell granules, leading to a characteristically dark appearance of the eggs in transmitted light (Fig. 1). The knock-down ovaries developed normally, reaching normal shape and size, but contained no granules in the growth zone (Fig. 6b and d). Oogonia and early oocytes I were still present in the ovary, but differentiation did not proceed into mature eggs. Gene annotation analysis of RNA815_2640 identified similarities with CPEB proteins involved in oocyte maturation in the clam *Spisula solidissima* (AAD12246.1) and the frog *Xenopus laevis* (NP_001089420) (see also Additional file 4). These proteins function as translational regulators of maternal mRNAs during meiotic progression, an important control mechanism in oocyte development [63]. The arrest of oocyte maturation observed in RNA815_2640 knock-down worms probably resulted from the suppression of CPEB mediated translational control during early meiosis I.

#### The knock-down of RNA815_80.4 prevents the AB staining of the prostate glands

The tail-specific transcript RNA815_80.4 was specifically expressed in the prostate glands (Figs. 3q and 7c-d). The knock-down worms showed the usual size, shape and distribution of the prostate glands (Fig. 7e) and a normal accumulation of secretion granules was observed in the gland bodies and necks, in the vesicula granulorum and in the central region of the stylet (Fig. 7e). Despite this apparently normal phenotype, immunocytochemical staining with the prostate-specific monoclonal antibody MPr-1 [58] revealed that no signal could be detected in the prostate glands of knock-down worms (Fig. 7b). In contrast, in the control worms (Fig. 7a), the antibody stained the prostate glands surrounding the seminal vesicles (while also unspecifically cross-reacting with the shell and cement glands around the female antrum, as is also detectable in knock-down worms). It is unclear if RNA815_80.4 directly codes for the protein targeted by the antibody or if the knock-down disrupted the specific pathway ultimately resulting in the synthesis of the protein containing the antibody's epitope. Sequence annotation analysis of RNA815_80.4 (Additional file 4) identified two WSC carbohydrate binding domains, but was uninformative on its possible specific function. This might be expected for a prostate gland or seminal fluid gene, as these genes are often evolving very rapidly (e.g. [32]).

## Discussion

The RNA-Seq dataset presented here allows us to identify genes exerting their function in single body regions that largely correspond to specific reproductive organs (i.e. the testes, ovaries and genital organs of the tail) in a non-model organism, the free-living flatworm, *M. lignano*. In combination with the simple RNAi protocol available in this species, these transcripts are promising targets for phenotypic engineering in order to manipulate sex-specific traits in evolutionary studies. Here we first discuss the features and predictive potential of our transcriptomic data. We then outline possible applications of the knock-down phenotypes identified in this study, which touch on a range of interesting aspects of the reproductive and evolutionary biology of *M. lignano*.

### Positional transcriptome

The primary goal of our study was to identify candidate genes for phenotypic engineering by generating a region-specific transcriptome. Given that we did not produce replicate samples of the different body fragments in our transcriptomic analysis, the positional classification of the transcriptome has to be considered with some care, because the lack of (biological or technical) replication does not allow for strict statistical comparisons to be made. However, our approach clearly permits an informed selection of candidates of interest within the transcriptome, provided that the expression specificity is further confirmed by ISH. Indeed, we found a very good agreement between the differential expression data and the ISH patterns for all candidates investigated here, thus clearly confirming that the dataset has a considerable predictive potential.

Some features of our positional transcriptome are probably attributable to technical issues related to our sampling strategy. Due to their small size, the worms were cut only once and every sample (other than the head-only fragment A) contained tissues common to other samples. Therefore, any apparent differential expression based on a pattern of declining expression level with increasing fragment size probably does not reflect real biological differences between samples, but can rather be attributed to a dilution effect due to the consecutive addition of novel transcript species in the adjacent fragments. Moreover, slight cutting errors and resulting contamination with small amounts of the ‘wrong’ tissues between samples were probably inevitable. For example, the egg shell/cement gland-specific transcript RNA815_13 is expressed in the very proximity of the third cutting level (between the ovaries and the tail, Fig. 1) and shows a quite large difference in expression between samples C and B (Table 1). A contamination of some tail-specific tissues into sample C would likely cause this pattern. Conversely, the three tail-specific candidates were expressed in the prostate glands, and thus far away from that cutting site; their expression level was therefore low or absent in samples A, B and C, meaning they could unambiguously be identified as tail-specific from the RNA-Seq data (Table 1). Finally, given the spatial proximity of the testes and the ovaries (Fig. 1), cutting errors at this level probably resulted in some contamination of sample B with ovarian tissues. As a consequence, transcripts specifically expressed in the ovaries, beside showing differential expression between samples C and B, might also show sufficiently high expression in sample B to reach our 2-fold threshold between sample B and A. The identification of five ovary specific transcripts in the [+,+,0] class suggests that many ovary-specific transcripts might fall into this class. Arguably, transcripts expressed in both gonads might be identified by setting a less stringent differential expression threshold between samples C and B.

Among the differentially expressed transcripts, the great majority had a testis-region specific expression (class [+,0,0]: 72.4 %), thus exceeding by almost an order of magnitude the transcripts with ovary region-specific expression. This pattern is consistent with the observation that testis transcriptomes show high levels of complexity in a range of organisms, including *D. melanogaster* [64], as well as in birds and mammals [65]. In mammals, the testes express more protein-coding and non-coding RNAs, splicing isoforms, and duplicated genes than other organs [65]. Such a phenomenon has been ascribed to the particularly open chromatin state of spermatogenic cells, resulting in high transcription activity [66, 67]. These features of testis gene expression are thought to reflect the selective pressure of post-copulatory sexual selection and sexual conflict on testicular function [68, 69]. Given that *M. lignano* is a simultaneous hermaphrodite, our results seem to suggest that this phenomenon is not restricted to species with separate sexes. It is possible that the large number of testis-specific transcripts identified in *M. lignano* reflects the functional complexity of the testes and is required for producing the highly elaborate sperm, which probably represent an adaptation to sperm competition and post-copulatory female choice (see below).

The positional classification of the transcriptome of *M. lignano* represents a highly useful resource for the rapidly growing *Macrostomum* research community. For example, it will permit the identification of conserved and non-conserved reproductive genes in related species, such as in *Macrostomum hystrix*, a species with hypodermic insemination, much simpler sperm and facultative self-fertilization [70, 71] and whose draft genome is currently also being sequenced (E. Berezikov, S. Ramm and L. Schärer, unpublished data). In the planarian *S. mediterranea*, very few ovary-specific markers have been identified, possibly due to the paucity and restricted distribution of ovarian tissues relative to testes [61, 72]. In *M. lignano*, this has previously limited the number of ovary-specific candidates for phenotypic engineering identifiable by homology. Our approach overcame this limitation by directly sequencing mRNAs from ovarian tissues and provided several novel ovary-specific candidates. Our transcriptomic data therefore represent a valuable comparative resource for studies of flatworm evolution, reproduction and development.

The capability to distinguish between organ-specific transcripts also is a powerful resource to investigate the physiology and behaviour of *M. lignano*. For example, recent progress in bio-adhesion research in *M. lignano* has resulted from a large-scale ISH screen of most of the tail-specific candidates identified in our study [73]. These screens have revealed a large number of genes with tail specific expression (>150) with a variety of expression patterns, some of which display adhesive-organ specific expression. Intriguingly, a strikingly large proportion of transcripts in these screens showed prostate-gland specific expression – as observed for RNA815_80.4 in this study-and these are currently being studied in the context of sexual selection and sexual conflict (S. Ramm, personal communication; see also below).

Moreover, an RNA-Seq analysis in worms raised in different group sizes-a condition that has previously been shown to induce a phenotypically plastic response in many sex-related traits [39, 43, 74, 75]-has recently been conducted (Ramm et al. in prep). This work revealed which parts of the transcriptome are particularly responsive to changes in the social environment. In combination with our positional RNA-Seq data, these data can be partitioned to identify which features of gene expression in the different reproductive functions underlie socially-induced plasticity.

In the present work we examined the potential of our approach to identify candidate transcripts in order to generate knock-down phenotypes for further experimental applications. To this end we identified several organ-specific RNAi phenotypes. Although we cannot unambiguously ascribe a direct causal link between the observed phenotypes and the candidate knock-down (as this would require reproducing the same phenotypes with non-overlapping dsRNA probes), a more detailed description of gene function was beyond the scope of the present study. In the next section we discuss the opportunities offered by the phenotypes identified here for the study of different aspects of the reproductive and evolutionary biology of *M. lignano*.

### Applications for phenotypic engineering in *M. lignano*

#### Neuropeptide signalling

In flatworms, there is growing evidence for a role of neuropeptide signalling in the regulation of a broad range of physiological functions [76–78]. In *S. mediterranea,* prohormone processing mediated by *Smed pc2* is required for the generation and maintenance of functional testes [61]. The severe effects of the knock-down of RNA815_2403.2 (homologous to *Smed pc2*; Additional file 4) on hatchlings suggest a relevant role of neuropeptide signalling in *M. lignano* postembryonic development. RNAi in adult worms might elucidate the possibility of a role of RNA815_2403.2 in reproductive function maintenance.

#### Sperm morphology and function

The highly complex morphology of the sperm of *M. lignano* [40, 42, 62] has been proposed to represent an adaptation to the high levels of post-copulatory sexual selection and sexual conflict that the mature sperm experience after being transferred to the female antrum of the partner [40, 42, 71, 75]. After copulation, a worm often performs a sucking behaviour over its own female genital opening. The lateral bristles in the sperm have been proposed to hinder sperm removal or rearrangement during this behaviour [42], a hypothesis that finds support in a comparative study among several species of the genus *Macrostomum* [71]. The inverted direction and the high flexibility of the bristles of the knock-down sperm under RNA815_7008 RNAi might reduce their ability to counteract the sucking behaviour, offering an elegant functional approach to test this hypothesis.

#### Sex allocation trade-off

In simultaneous hermaphrodites, sex allocation refers to the resource allocation to the two sexual functions (reviewed in [79]) and *M. lignano* has been extensively used in the study of sex allocation in simultaneous hermaphrodites (e.g. [43, 44, 80, 81]). Our study identified an ovary-specific transcript (RNA815_2640) whose RNAi knock-down suppresses egg production and thereby possibly reduces resource allocation to the female function. In a phenotypic engineering study of ovarian function (Sekii et al. in prep.), the knock-down of RNA815_2640 resulted in a significant increase in testis size and in a trend towards increased sperm production rate. This study provided direct experimental evidence of a resource allocation trade-off between sexual functions, an important assumption of sex allocation theory [79], nicely illustrating the usefulness of the data we have generated here.

#### Prostate gland function

In an increasing number of species prostate gland secretions (or seminal fluids) that are transferred during copulation have been shown to manipulate the physiology and behaviour of the partner, in a way that increases the sperm donor’s reproductive success, sometimes at a cost to the receiver [82–84]. Such a role for prostate secretions has, for example, been demonstrated in *D. melanogaster* [19, 85] and in some simultaneously hermaphroditic snails [86–88]. Moreover, in promiscuous simultaneous hermaphrodites with obligate reciprocal mating, high levels of post-copulatory sexual selection and sexual conflict are expected to occur [42, 89–91]. This suggests a possibly important role for prostate secretions in partner manipulation and the evolution of mechanisms to counteract such manipulations [89, 90, 92]. Our study provides ground for the functional characterization of prostate gland secretions and will help to elucidate their role in post-copulatory sexual selection and sexual conflict in *M. lignano*.

## Conclusions

Many emerging model organisms are specifically chosen (i) as representatives of currently poorly investigated taxa and/or (ii) due to some unique aspects of their biology, both of which will often mean that functional approaches are restricted due to a lack of suitable candidate genes. Here we have shown how-in the emerging flatworm model species *M. lignano-*a combination of sequencing resources (i.e. a transcriptome and positional RNA-Seq) and functional analysis tools (i.e. ISH and RNAi) can provide an efficient approach for identifying candidate genes for phenotypic engineering studies. By directly sequencing tissues likely to generate phenotypes of interest (i.e. traits related to specific reproductive functions), we could identify several promising candidate genes, therefore overcoming the current lack of exhaustive functional sequence annotation in our model organism. Given the opportunity offered by progress in sequencing technologies and the advances in RNAi knock-down and genome editing tools, we expect that our approach will help to bring phenotypic engineering to many emerging model organisms.

## Methods

### Study organism

The free-living flatworm *Macrostomum lignano* (Lophotrochozoa: Platyhelminthes: Macrostomorpha) is an outcrossing simultaneous hermaphrodite and member of the interstitial sand fauna of the Northern Adriatic Sea [37]. Adults reach 1.5 mm in body length, and their overall anatomical organization is very distinct, with the male and female gonads distributed along the body axis on both sides of a central gut, and the male and female genital organs in the tail (Fig. 1). The worm’s transparency permits the observation of internal organs and processes (e.g. spermatogenesis) and the non-invasive observation of reproductive structures *in vivo*. In the laboratory, worms are maintained in mass cultures in glass Petri dishes filled with 20 ml of nutrient-enriched artificial seawater (f/2 medium, [93]) and fed with diatoms (*Nitzschia curvilineata*). The dishes are kept in a climate-chamber with a 14:10 day-night cycle, 60 % humidity and constant temperature of 20 °C [94]. All the animals analysed here belonged to the DV1 inbred line [80] used to generate the reference genome and transcriptome [56, 57]. All animal experimentation was carried out in accordance to Swiss legal and ethical standards.

### RNA-Seq

For RNA-Seq, tissue samples were collected from several hundred adult worms (22 days post hatching), which were cut with a scalpel under a dissecting microscope. The worms were cut at one of three different levels – anterior to the testes, between testes and ovaries, or between ovaries and the female antrum – to obtain increasingly larger fragments, each with an additional body region, namely the head fragment (sample A), the head + testis fragment (sample B), the head + testis + ovary fragment (sample C), and finally whole worms (sample D) (Fig. 1). The different fragments were cut over several days in a repetitive, systematic order to avoid any sequence effects, collected in TRI reagent® immediately after cutting, and subsequently pooled into 4 separate samples designated A, B, C, and D. Total RNA was extracted with TRIzol® Reagent (Ambion) and processed to generate four non-directional single-read Illumina RNA-Seq libraries. The libraries were sequenced with 36 cycles on an Illumina Genome Analyzer II, following the manufacturer’s protocols. The sequencing reads generated were approximately 48 × 10^6^ (Sample A), 47 × 10^6^ (Sample B), 45 × 10^6^ (Sample C), and 55 × 10^6^ (Sample D).

### Bioinformatic analysis

The unfiltered sequencing reads (36 bp) were mapped to the *M. lignano de novo* transcriptome assembly (version MLRNA110815; [56, 57] using Bowtie software v 0.12.7 (PMID: 19261174) with the parameters “-m 200-best-strata”. The resulting sam files were parsed to exclude hits with more than 5 mismatches and further processed by RSEM software v. 1.1.10 (PMID: 21816040) to estimate the read counts per transcript taking into account multiple mapping. Read counts were further normalized to yield a measure of the expression level, as follows:

$$ Expression\  level = { \log}_2\left[\left(\frac{Transcript\  read\  count}{Total\  mapped\  read s\  in\  sample}\ *100\right)+0.00001\right] $$

The expression levels of all the transcripts were compared by calculating the difference in expression between adjacent samples, with the most informative comparisons being the difference between samples B and A (effect of adding the testis fragment), C and B (effect of adding the ovary fragment) and D and C (effect of adding the tail fragment), respectively (Fig. 2). We considered the expression of a transcript to be different if the log2-expression level between adjacent samples varied more than two-fold (corresponding to a four-fold threshold on a linear scale). This threshold is to some extent arbitrary and motivated by the exploratory nature of our analysis, considering that false positives could be later detected by *in situ* hybridization. Positive fold changes >2 between adjacent samples indicate higher expression in the larger fragment, while negative values are thought to reflect a dilution effect attributable to the consecutive addition of novel transcript species in additional fragments (see Discussion). A differential expression profile that considers all comparisons between adjacent samples was assigned to each transcript: it represents a three digit code, where each digit refers to the fold-change in expression level between two adjacent samples ([B-A], [C-B], [D-C]) with three possible values: “+” (fold-change > 2), “0” (2 > fold-change > −2) or “-“(fold-change < −2). Thus, transcripts could be classified by their differential expression profile (further called a ‘class’), allowing us to predict their site of expression: non-differentially expressed transcripts (class [0,0,0]), testis region-specific transcripts (class [+,0,0]), ovary region-specific transcripts (class [0,+,0] and class [+,+,0]), tail region-specific transcripts (class [0,0,+]) and transcripts with complex differential expression patterns (other classes) (see also Table 1).

### Whole mount *in situ* hybridization screening

23 candidate transcripts were selected for an *in situ* hybridization (ISH) screen. We included transcripts that were predicted to be either non-differentially expressed (n = 4), testis region-specific (n = 6), ovary-specific (n = 8) and tail-specific (n = 3) (see above). Moreover, two additional candidates with other differential expression patterns were also chosen. In order to remain unbiased, transcripts were selected independently of their annotation in the transcriptome assembly. Forward and T7-reverse primer pairs were designed for each candidate transcript with Primer3 version 0.4.0 [95] to attain optimal probe length (about 600 bp; Additional file 3). cDNA was synthesized from total RNA from adults worms from a mass culture, using the High Capacity RNA-to-cDNA Kit (Applied Biosystems) and amplified with transcript specific primer pairs. PCR conditions were: 94 °C 2 min, (94 °C 30 s, 60 °C 30 s, 72 °C 1 min 30 s) × 10, (94 °C 30 s, 55 °C 30 s, 72 °C 1 min 30 s) × 20, 72 °C 7 min. PCR products were used to synthesize single stranded anti-sense DIG-labeled RNA probes with the DIG RNA Labelling KIT (Roche). ISH was performed according to Pfister et al. [47], using 10–15 adult animals in every reaction. The signal was developed at room temperature using the NBT/BCIP system (Roche). Micrographs of the specimens were taken under brightfield and interference contrast illumination with a Leica DM2500 compound microscope (Leica Microsystems), a digital video camera (DFK 41BF02, The Imaging Source, Europe) and the software BTV Pro 6.0b1 (Bensoftware).

### RNA interference screen

The synthesis of dsRNA was performed using the T7 RiboMAX™ Express Large Scale RNA Production System (Promega). cDNA was amplified using T7 primer pairs (Additional file 3) for each candidate transcript (PCR conditions as before). A DNase treatment was performed using the RQ1 RNase-Free DNase (Promega). For controls, the firefly luciferase sequence was cloned into a plasmid (pGEM®-luc Vector from Promega) and amplified by PCR using specific primers (LucFw: GTCTTTCCGTGCTCCAAAAC; LucRev: CCAGGGATTTCAGTCGATGT). The rationale of using this control gene, which does not exist in *M. lignano*, is to control for any unspecific effects of exposure to dsRNA. The PCR product was purified from an agarose gel using the QIAquick Gel Extraction (QIAGEN) following the manufacturer’s instructions and used for dsRNA synthesis. All 23 transcripts were screened with an RNAi-by-soaking method, as previously described [54, 55].

15 worms for each candidate transcript were included in the screening. Treatments started from the first day post-hatching and were maintained until the worms reached sexual maturity or died. The worms were kept individually in 10 μl of a solution of double stranded RNA in autoclaved artificial sea water (ASW) (dsRNA concentration: 4 ng/μl) with antibiotics (antibiotic concentration: 50 μg ml^−1^), randomized in 60-well microtest plates (Greiner Bio One) and fed with algae *ad libitum* in normal laboratory conditions. Kanamycin or ampicillin were alternated every second day to prevent the selection of resistant bacterial strains. Control worms were maintained in the same conditions in autoclaved ASW or in a solution of firefly luciferase dsRNA (dsRNA concentration: 4 ng/μl). No differences between the ASW and the luciferase dsRNA control treatments could be detected. The worms were transferred every day to fresh solution to ensure exposure to constant dsRNA concentration. The phenotypes were documented *in vivo* (see above), after carefully squeezing the worms between a microscope slide and a cover slip. At the end of the screen, the efficiency of transcript knock-down in the experimental animals was assessed by ISH using 3 to 5 animals per candidate transcript.

To further assess the phenotype of the RNAi knock-down of three tail region-specific candidate transcripts, we performed immunocytochemical staining on RNAi-treated and control worms (n = 3 for each) as previously described [58] using a prostate-specific monoclonal antibody (MPr-1) and a secondary FITC-conjugated goat anti-mouse antibody (Dako). Micrographs were taken with the same compound microscope under epifluorescence, using a Leica DFC945 digital video camera and the Leica Application Suite V3.3.

### Sequence annotation analysis

Sequence annotation was performed by using BLAST search (blastx) against available protein databases [96].

### Availability of supporting data

The data set supporting the results of this article is available in the Sequence Read Archive (SRA) repository, SRP052579, http://www.ncbi.nlm.nih.gov/sra?term=SRP052579.

## Additional files

Additional file 1:
***Macrostomum lignano*** **positional transcriptome.** (**A**) Transcript ID in transcriptome version MLRNA110815 [56, 57]; (**B**-**E**) RNA-Seq reads mapped in samples **A**-**D**; (**F**-**I**) Transcript expression level in samples **A**-**D**; (**J**-**L**) Difference in transcript expression level between samples B-A, C-B and **D**-**C**; (**M**) Transcript class; (**N**) Transcript length. See Methods for details on expression level quantification and classification. Additional file 2:
**Animation of the positional transcriptome of** ***Macrostomum lignano.*** The movie shows a rotation of the space of the differences in expression level between adjacent RNA-Seq samples (i.e. B vs. A, C vs. B and D vs. C). Additional file 3:
**Selected candidate transcripts.** Summary table of the candidate transcripts and their ISH/dsRNA probe sequences. (**A**) Transcript ID in transcriptome version MLRNA110815 [56, 57]; (**B**) Transcript class; (**C**) Transcript sequence; (**D**) Transcript length; (**E**) Forward primer sequence; (**F**) Reverse primer sequence; (**G**) ISH/dsRNA probe length. Additional file 4:
**Candidate transcripts homology.** Results of the homology search (blastx) for the candidate transcripts against the non-redundant protein database (NCBI, date of the analysis: 01.11.2014). (**A**) Transcript ID in transcriptome version MLRNA110815 [56, 57]; (**B**) Best ten blastx hit accession number; (**C**) Best ten blastx hit description [Species]; (**D**) E-value, (**F**) Positives (%). Blank entries indicate that no clear homology was found for a given candidate transcript. Additional file 5:
**Summary of the RNAi screen for RNA815_2403.2, RNA815_7008, RNA815_9973.1, RNA815_2640, and RNA815_80.4.** (**A**) Transcript ID in transcriptome version MLRNA110815; (**B**) Brief description of the RNAi phenotype (s); (**C**) Number of worms showing each RNAi knock-down phenotype and total number of worms assayed; (**D**) Expression specificity of the candidates; (**E**) Number of worms showing expression of each candidate after RNAi knock-down and total number of worms assayed. Additional file 6:
***In situ*** **hybridization (ISH) of control and RNAi knock-down worms.** (**A**) RNA815_7008 control; (**B**) RNA815_7008 knock-down; (**C**) RNA815_9973.1 control; (**D**) RNA815_9973.1 knock-down; (**E**) RNA815_2640 control; (**F**) RNA815_2640 knock-down; (**G**) RNA815_80.4 control; (**H**) RNA815_80.4 knock-down.
