# Supplementary figures and images for: Positional RNA-Seq identifies candidate genes for phenotypic engineering of sexual traits
Source: Front Zool. 2015 Jul 3;12:14. doi: 10.1186/s12983-015-0106-0 (PMC4490696; doi:10.1186/s12983-015-0106-0)

## Control

## Knock-down

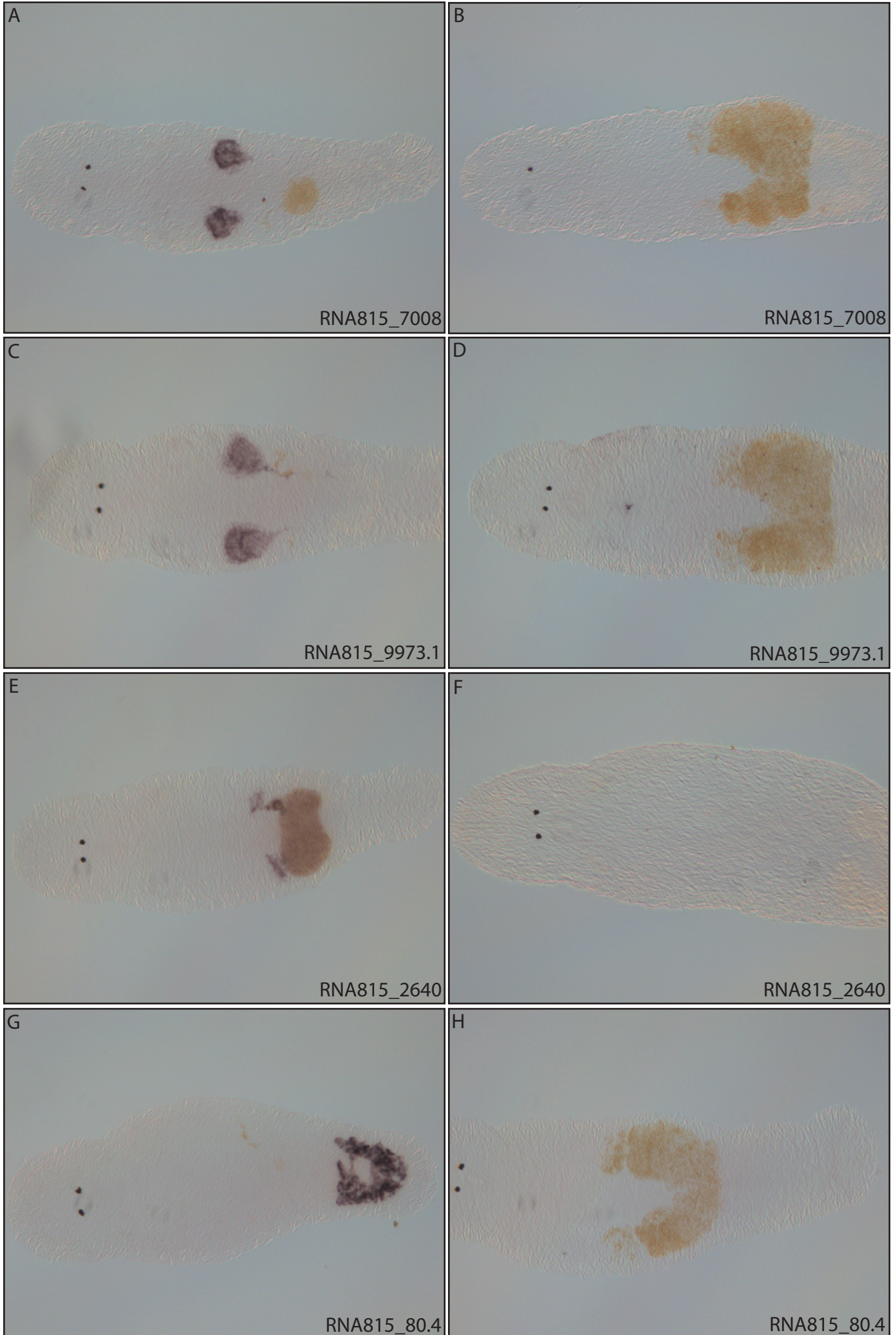

Supplement: Additional file 6: — In situ hybridization (ISH) of control and RNAi knock-down worms. (A) RNA815_7008 control; (B) RNA815_7008 knock-down; (C) RNA815_9973.1 control; (D) RNA815_9973.1 knock-down; (E) RNA815_2640 control; (F) RNA815_2640 knock-down; (G) RNA815_80.4 control; (H) RNA815_80.4 knock-down. [file 12983_2015_106_MOESM6_ESM.pdf]
